# STAT3/HIF-1α signaling activation mediates peritoneal fibrosis induced by high glucose

**DOI:** 10.1186/s12967-021-02946-8

**Published:** 2021-06-30

**Authors:** Xiaoxiao Yang, Manchen Bao, Yi Fang, Xiaofang Yu, Jun Ji, Xiaoqiang Ding

**Affiliations:** 1 Department of Nephrology, Zhongshan Hospital, Fudan University, No. 180, Fenglin Road, Xuhui District, Shanghai, 200032 People’s Republic of China; 2 Shanghai Medical Center of Kidney, Shanghai, China; 3 Shanghai Institute of Kidney and Dialysis, Shanghai, China; 4 Shanghai Key Laboratory of Kidney and Blood Purification, Shanghai, China; 5 Hemodialysis Quality Control Center of Shanghai, Shanghai, China

**Keywords:** Peritoneal dialysis, Peritoneal fibrosis, STAT3, HIF-1α, Epithelial-mesenchymal transition

## Abstract

**Background:**

Epithelial-mesenchymal transition (EMT) of mesothelial cells is a key step in the peritoneal fibrosis (PF). Recent evidence indicates that signal transducer and activator of transcription 3 (STAT3) might mediate the process of renal fibrosis, which could induce the expression of hypoxia-inducible factor-1α (HIF-1α). Here, we investigated the effect of STAT3 activation on HIF-1α expression and the EMT of mesothelial cells, furthermore the role of pharmacological blockade of STAT3 in the process of PF during peritoneal dialysis (PD) treatment.

**Methods:**

Firstly, we investigated the STAT3 signaling in human peritoneal mesothelial cells (HPMCs) from drained PD effluent. Secondly, we explored the effect of STAT3 signaling activation on the EMT and the expression of HIF-1α in human mesothelial cells (Met-5A) induced by high glucose. Finally, peritoneal fibrosis was induced by daily intraperitoneal injection with peritoneal dialysis fluid (PDF) so as to explore the role of pharmacological blockade of STAT3 in this process.

**Results:**

Compared with the new PD patient, the level of phosphorylated STAT3 was up-regulated in peritoneal mesothelial cells from long-term PD patients. High glucose (60 mmol/L) induced over-expression of Collagen I, Fibronectin, α-SMA and reduced the expression of E-cadherin in Met-5A cells, which could be abrogated by STAT3 inhibitor S3I-201 pretreatment as well as by siRNA for STAT3. Furthermore, high glucose-mediated STAT3 activation in mesothelial cells induced the expression of HIF-1α and the profibrotic effect of STAT3 signaling was alleviated by siRNA for HIF-1α. Daily intraperitoneal injection of high-glucose based dialysis fluid (HG-PDF) induced peritoneal fibrosis in the mice, accompanied by the phosphorylation of STAT3. Immunostaining showed that phosphorylated STAT3 was expressed mostly in α-SMA positive cells in the peritoneal membrane induced by HG-PDF. Administration of S3I-201 prevented the progression of peritoneal fibrosis, angiogenesis, macrophage infiltration as well as the expression of HIF-1α in the peritoneal membrane induced by high glucose.

**Conclusions:**

Taken together, these findings identified a novel mechanism linking STAT3/HIF-1α signaling to peritoneal fibrosis during long-term PD treatment. It provided the first evidence that pharmacological inhibition of STAT3 signaling attenuated high glucose-mediated mesothelial cells EMT as well as peritoneal fibrosis.

**Supplementary Information:**

The online version contains supplementary material available at 10.1186/s12967-021-02946-8.

## Introduction

Peritoneal dialysis (PD) has been a well-established therapeutic modality for patients with end-stage renal disease (ESRD). Its efficacy depends on both the structural and functional integrity of peritoneal membrane. Acquired peritoneal fibrosis (PF) is a major factor leading to ultrafiltration failure and finally treatment interruption in PD patients. The condition is thought to occur in response to a variety of insults, including continuous exposure to bioincompatible dialysate (acidic solution, high glucose, glucose degradation products, low pH, high osmolality), uremic toxins, and peritonitis [1–3]. Among these components, high glucose has been widely regarded as a key factor contributing to structural and functional alterations of the peritoneal membrane [4].

The process of peritoneal fibrosis includes the loss of mesothelial cells, abnormal proliferation of α-smooth muscle actin (α-SMA)-positive fibroblasts, and thickening of sub-mesothelial area with accumulation of collagen [5–7]. Epithelial-mesenchymal transition (EMT) of human peritoneal mesothelial cells (HPMCs) is a key step in the PF. At its worst, peritoneal injury leads to peritoneal sclerosis, a serious complication, which induces technique failure and high mortality in PD patients. In parallel with these alterations, the peritoneal membrane undergoes typical functional change-increased peritoneal small-solute transport rate (PSTR), leading to impaired ultrafiltration and ultimately discontinuation of treatment [8]. Thus, to maintain long-term treatment and improve high quality of life for PD patients, it is important to develop interventions for the prevention and treatment of the PF.

Signal transducer and activator of transcription 3 (STAT3) mediates various cellular functions, including cell survival and proliferation [9, 10]. In response to numerous growth factors and cytokines, STAT3 is activated by tyrosine phosphorylation at tyrosine 705 through Janus kinase. Phosphorylated STAT3 forms a dimer and then transfers to the nucleus where the dimer directly binds to DNA sequence and regulates the expression of target genes [11–13]. In recent years, we have reported that interleukin-6 (IL-6) drives a STAT3-dependent pathway that leads to structural and functional alterations of peritoneal membrane in a mouse peritoneal fibrosis model [14]. It has been reported that high glucose directly induced STAT3 phosphorylation of renal tubular epithelial cells and pharmacological inhibition of STAT3 attenuated the progression of diabetic nephropathy [15]. In line with that, it has been also reported that STAT3 inhibitor could reduce transforming growth factor-β1(TGF-β1) expression and alleviate the EMT process of mesothelial cells induced by high glucose [16]. However, the effect of STAT3 inhibitor on the progression of peritoneal fibrosis in vivo is still not clear.

STAT3 has been reported to induce the expression of multiple genes [11–13]. It has reported that the expression of TGF-β1, angiotensin II generating enzyme (ACE) or angiotensin II receptor type 1 (AT1), and vascular endothelial cell growth factor (VEGF) of renal tubular epithelial cells induced by high glucose could be abrogated by STAT3 inhibition [15]. Furthermore, it has been reported that interleukin-37 (IL-37) suppressed hypoxia-inducible factor-1α (HIF-1α) expression through STAT3 inhibition during the progression and chemoresistance of pancreatic cancer [17]. It is well known that HIF-1α is a key regulator of cell response to hypoxia [18]. Chronic hypoxia in renal interstitium is one of the characteristics during the progression of chronic kidney disease (CKD), which can lead to tubular cell EMT or apoptosis, activate resident fibroblast [19]. Morishita et al. [20] reported that hypoxia induced the EMT in mesothelial cells (Met-5A) via up-regulation of HIF-1α, while HIF-1α inhibition could attenuate this process. Under this background, we speculated that there might be a link between STAT3 activation and HIF-1α expression, involving the EMT process of mesothelial cells induced by high glucose.

Thus, in this study, we investigated the effect of STAT3 activation on HIF-1α expression and the EMT of mesothelial cells induced by high glucose, as well as the role of pharmacological blockade of STAT3 in the process of PF during PD treatment.

## Materials and methods

### Ethics statement

The study protocol conformed to the ethical guidelines of the 1975 Declaration of Helsinki and was approved by the Ethic Committee of Zhongshan Hospital. Written informed consent was obtained from each participant. The experimental protocols strictly complied with the institutional guidelines and the criteria outline in the Guide for the Care and Use of Laboratory Animals (NIH Publication No. 80-23) and approved by Zhongshan Hospital, Fudan University.

### Materials

We obtained antibodies targeting p-STAT3 (CST, #9145), STAT3 (CST, #4904), E-cadherin (Proteintech, #20874), Fibronectin (Proteintech, #15613), HIF-1α (Proteintech, #2096), GAPDH (Proteintech, #10494), Collagen I (Santa Cruz, sc-293182), α-SMA (Santa Cruz, sc-53142), CD31 (Santa Cruz, sc-376764), F4/80 (Santa Cruz, sc-377009). S3I-201 was purchased from Merck Life Science (S1155, Shanghai, China). M199 medium and other cell culture reagents were obtained from Gibco BRL. Glucose and mannitol were obtained from Sigma.

### HPMCs isolation and cell culture

PD effluent was obtained from PD patients after dwell times ranging from 7 to 10 h. The samples were processed immediately upon collection. All patients used PD solution from Dianeal Baxter company. HPMCs obtained from the effluent of PD patients in our center, were cultured and characterized as described in detail elsewhere [14, 21]. The exclusion criteria were as follows: presence of systemic inflammatory disease, peritonitis or fluid overload within the past 3 months, history of malignant tumor, taking high-dose glucocorticoid or immunosuppressive agents during the past year. The following demographic characteristics were collected at the enrollment of the study: age, gender, underlying cause of ESRD, PD duration.

The human mesothelial cell line (Met-5A: CRL-9444) was obtained from ATCC (Manassas, VA, USA) and cultured in M199 medium containing 5.5 mM of d-glucose supplemented with 10% FCS (Gibco), and 1% penicillin/streptomycin solution (Gibco) under sterile conditions of 5% CO_2_ at 37 °C. Before treatment, mesothelial cells were cultured in 60-mm plates overnight. In the high glucose-treated group, cells were incubated with a M199 medium containing 60 mM glucose. Mesothelial cells were incubated with the same concentrations of mannitol medium as described in our previous study [22]. The exact treatment of mesothelial cells was described in the corresponding figure legends.

### Establishment of peritoneal fibrosis mouse model and S3I-201 administration

The male C57BL/6J mice (8 to 10 weeks old) were housed under specific pathogen free conditions at Laboratory Animal Center in Zhongshan Hospital, Fudan University. Animals were divided into three groups (n = 5): the sham group, the peritoneal fibrosis group induced by high glucose based peritoneal dialysis fluid (HG-PDF) with or without S3I-201.The same as previous studies [23, 24], peritoneal fibrosis was induced by daily intraperitoneal injection with 100 mL/kg PDF containing 4.25% glucose. Mice in the sham group were injected with an equal volume of 0.9% saline, accordingly. To explore the effect of STAT3 inhibition on peritoneal fibrosis, S3I-201 was administered intraperitoneally at a dose of 10 mg/kg per mouse daily according to the previous studies [25, 26]. After 28 days, mice were euthanized and parietal peritoneum far from the injection points was harvested for further analysis. Peritonea were either fixed in 4% paraformaldehyde for pathological analysis or flash-frozen in liquid nitrogen for gene and protein expression analysis.

### Reverse transcription and real-time quantitative PCR

Total RNA was isolated from cells and tissues using TRIZOL (Invitrogen, Carlsbad, CA) according to the manufacturer's instructions. Total RNA was reverse-transcribed to cDNA using PrimeScript RT Master Mix kit (Takara). Real-time PCR reaction was performed in triplicate using SYBR Premix Ex Taq II (Tli RNaseH Plus, Takara) and analyzed with a 7500 real-time PCR system (Thermo Fisher Scientific). All primers were purchased from Sangon Biotech (Shanghai, China). Relative changes in mRNA were calculated using ΔΔCt method and standardized to housekeeping gene GAPDH.

Mouse genes and primers:

*MCP-1*, forward, 5'-TTTTTGTCACCAAGCTCAAGAG-3',

reverse, 5'-TTCTGATCTCATTTGGTTCCGA-3'.

*IL1β*, forward, 5'-TCGCAGCAGCACATCAACAAGAG-3',

reverse, 5'-AGGTCCACGGGAAAGACACAGG-3'.

*Col1a1*, forward, 5-TAAGGGTCCCCAATGGTGAGA-3'.

reverse, 5'-GGGTCCCTCGACTCCTACAT-3'.

Acta2, forward, 5'-CCCAGACATCAGGGAGTAATGG-3'.

reverse, 5'-TCTATCGGATACTTCAGCGTCA-3'.

*Hif1a*, forward, 5'- GATGAGTTCTGAACGTCGAAAAG-3'.

reverse, 5'- CACTGTCTAGACCACCGGC-3'.

*Tgfb1*, forward, 5'- CTCCCGTGGCTTCTAGTGC-3'.

reverse, 5- GCCTTAGTTTGGACAGGATCTG-3'.

### Immunoblot analysis

Total protein was extracted from tissues or cultured cells using RIPA buffer (Beyotime, Shanghai, China). Protein concentrations were determined using Pierce BCA Protein Assay Kit (Thermo Fisher). Proteins (20–50 μg) were boiled, separated on SDS–polyacrylamide gel electrophoresis, and transferred to PVDF membranes (Bio-Rad). Membrane was blocked with 5% bovine serum albumin in TBS with 0.1% Tween 20 (Sigma Aldrich) for 1 h at room temperature and incubated overnight at 4 °C with primary antibodies. The bands obtained were visualized and analyzed using the Enhanced Chemiluminescence Detection System (Tanon Science & Technology Co., Ltd) and Image J 1.43 software (National Institute of Health, Bethesda, MD). The data were normalized against GAPDH.

### Histological analysis of the peritoneum

Peritonea were fixed in 4% paraformaldehyde and then embedded in paraffin and prepared in 5 µm thick sections. To evaluate the peritoneal fibrosis, Masson trichrome staining was performed according to the protocol provided by the manufacturer (Sigma-Aldrich).

Paraffin-embedded sections (5 µm thick) were deparaffinized and rehydrated. Then antigen was retrieved at 98 °C for 10 min in 10 mM citrate buffer with pH 6 and washed with phosphate-buffered saline (PBS) for 15 min, and then the sections were treated with blocking buffer containing 5% bovine serum albumin for 30 min at room temperature before the overnight incubation with primary antibodies at 4 °C. The following antibodies were used: Collagen I (Santa Cruz, sc-293182, 1:100), CD31 (Santa Cruz, sc-376764, 1:100), F4/80 (Santa Cruz, sc-377009, 1:200). After having washed with PBS three times, the secondary antibody was added and counterstaining hematoxylin was performed, and DAB positivity was analyzed in six visual fields at 200× magnification. The positive area was measured using Image J (National Institute of Health, USA).

### Immunofluorescence

Deparaffinized and rehydrated paraffin-embedded sections (5 µm thick) were prepared for antigen retrieval at 98 °C for 10 min in 10 mM citrate buffer with pH 6 and washed with PBS for 15 min and treated with blocking buffer containing 5% horse serum in PBS for 30 min at room temperature before the overnight incubation with the primary antibodies at 4 °C. Secondary labeled antibodies: Alexa Fluor®488 (Abcam, ab150077) or Alexa Fluor®594 (Abcam, ab150116) were incubated with peritoneum sections for 1 h at room temperature. Negative control for immunofluorescence staining was conducted using 5% horse serum instead of primary antibodies. Images were analyzed by computerized digital image analysis (AnalySIS, Soft Imaging System).

HPMCs and Met-5A cells were seeded with a density of 10^4^ cells/well and incubated with different stimulation as described in figures legends. Then cells were fixed with 4% paraformaldehyde, incubated with primary antibodies. The following antibodies were used: p-STAT3 (CST, #9145, 1:1000), E-cadherin (Proteintech, #20874, 1:1000), Fibronectin (Proteintech, #15613, 1:1000), Collagen I (Santa Cruz, sc-293182, 1:100), α-SMA (Santa Cruz, sc-53142, 1:200). The slides were incubated with Alexa Fluor®488 or Alexa Fluor®594 for 1 h, respectively. Nuclei were labeled with DAPI (Life Technology) and then slides were examined using fluorescence microscope.

### Transfection study in mesothelial cells

Silence of gene expression was achieved using siRNA specific target sequences. All of siRNA specific target sequences were obtained from HanBio (Shanghai, China).

Human siRNA sequence as following:

STAT3: sense, 5'- CGUCAUUAGCAGAAUCUCAtt-3',

antisense, 5'- UGAGAUUCUGCUAAUGACGtt-3'.

HIF-1α: sense, 5' -GCCGAGGAAGAACUAUGAATT-3',

antisense, 5'-UUCAUAGUUCUUCCUCGGCTT-3'.

Control: sense, 5'-UUCUCCGAACGUGUCACGUTT-3',

antisense, 5'-ACGUGACACGUUCGGAGAATT-3'.

Met-5A cells were transiently transfected with 100 nM target sequences or negative control (NC) siRNA using Lipofectamine 2000 (Invitrogen, CA, USA) according the manufacturer's instructions. Then transfected cells were treated with high glucose for subsequent experiments.

### Statistical analyses

The numeric data were presented as the mean ± standard error (SE) and were generated using SPSS 12.0 software (Chicago, IL). In this paper, each experiment was generally repeated for 5–6 times with different cells, and three of them were randomly selected for statistical analysis. We generally compared the difference among multiple groups using ANOVA. To compare the difference between each two groups, Kruskal–Wallis test was used for abnormal distributed data and t test for normal distributed data. Difference was considered to be significant at *P* < 0.05.

## Results

### STAT3 activation of mesothelial cells ex vivo in drained PD effluent

**Fig. 1:**
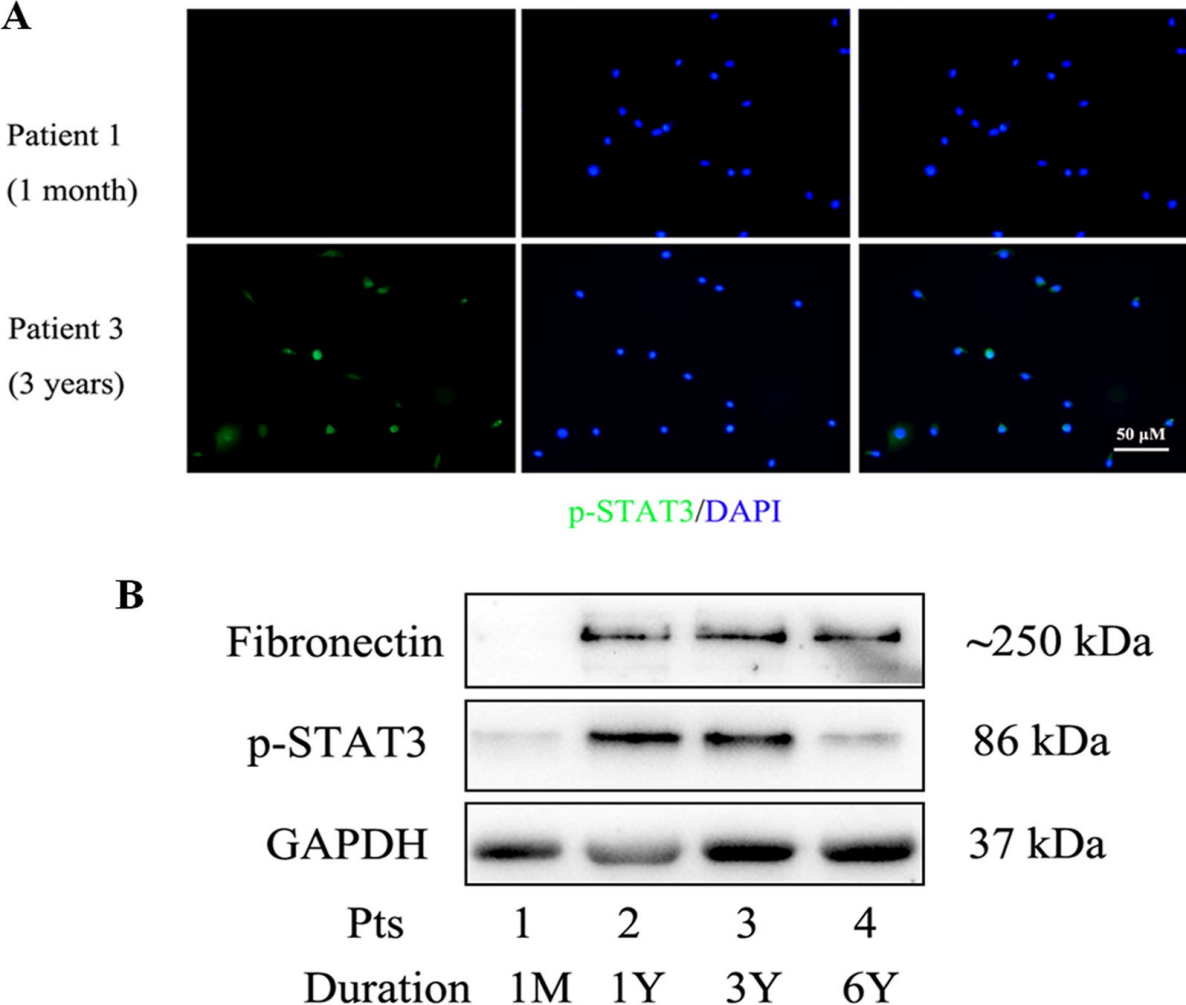
The level of phosphorylated STAT3 of HPMCs ex vivo in drained PD effluent. **A** HPMCs were cultured from drained PD effluent of two patients (patient 1: PD duration 1 month; patient 3: PD duration 3 years). Then cellular localization of p-STAT3 (green) in HPMCs was detected using immunofluorescence analysis. Original magnification, ×200. **B** Western blot was performed with PD effluent cell lysates centrifuged from patients with different PD duration. The levels of Fibronectin and p-STAT3 were present in these long-term patients (No. 2, 3, 4; PD duration 1 year, 3 years and 6 years, respectively) but not in a new patient (No. 1; PD duration 1 month)

We collected drained PD effluent from patients with different treatment duration. Patients' clinical summaries were shown in Additional file 1: Table S1. We firstly investigated the expression of p-STAT3 in cast-off human mesothelial cells of drained PDF by immunofluorescence. There was a significantly increasing expression of p-STAT3 in No. 3 patient (with 3 years PD duration) compared with No. 1 patient (with 1 month PD duration. Figure 1A). We further investigated the PD effluent cell lysates using western blot analysis. As shown in Fig. 1B, the levels of Fibronectin and p-STAT3 were significantly up-regulated in long-term patients (No. 2, 3, 4) but not in the new patient (No. 1). Taken together, these findings indicated that STAT3 pathway was activated in mesothelial cells from PD patients with long-term treatment.

### Effect of high glucose on STAT3 activation in mesothelial cells

**Fig. 2:**
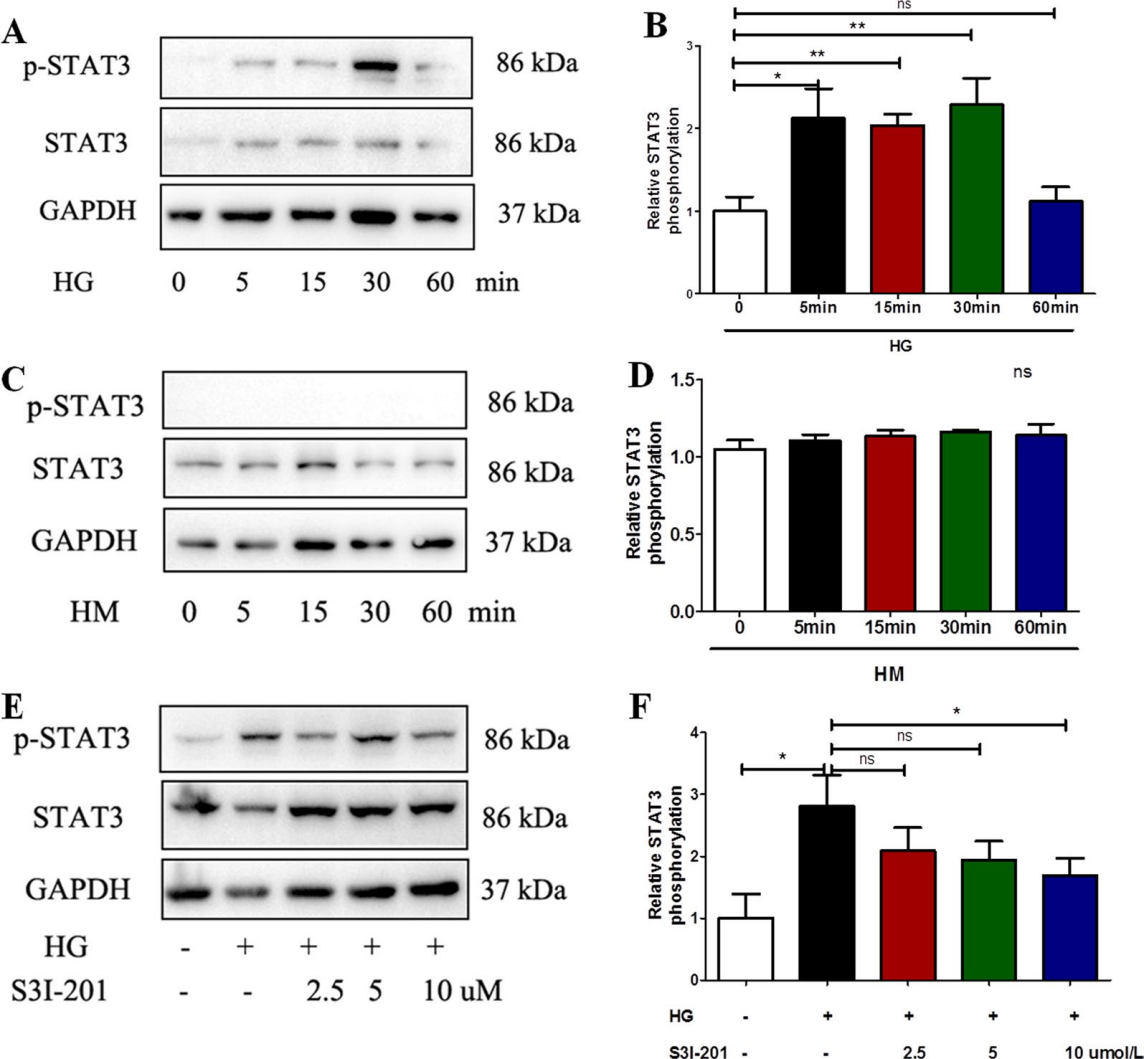
High glucose activated the STAT3 pathway in Met-5A cells. Cells were incubated with high glucose (60 mmol/L, **A**) or high mannitol (60 mmol/L, **C**) for different time (0, 5, 15, 30, 60 min). Western blot analysis was performed to detect the protein expressions of p-STAT3 and STAT3. The relative ratios of p-STAT3 to total STAT3 were expressed as the mean ± SE of three experiments (**B**). **D**, **E** Met-5A cells were incubated for 30 min with high glucose (60 mmol/L) or in combination with different concentrations of STAT3-specific inhibitor S3I-201(2.5, 5, 10 μM, pretreated for 1 h), and the relative ratios of p-STAT3 to total STAT3 were expressed as the mean ± SE of three experiments (**E**). *P* values were determined by t tests. *P < 0.05; **P < 0.01; ns. not significant

We then explored the effect of high glucose on STAT3 activation in mesothelial cells (Met-5A) using western blot analysis. The results showed that high glucose (60 mmol/L) induced STAT3 phosphorylation in Met-5A cells after incubating with 5 min and the effect was peak in 30 min, then disappeared in 60 min (*P*< 0.05 compared to baseline at 5, 15, 30 min, Fig. 2A, B); While mannitol (60 mmol/L) had no significant effect on STAT3 phosphorylation after incubation with the same time (Fig. 2 C-D). At the concentration of 10 μM, the STAT3 inhibitor S3I-201 (pretreatment for 1 h) abolished high glucose (60 mmol/L, incubated with for 30 min) induced STAT3 phosphorylation without affecting the total STAT3 expression in Met-5A cells (*P* < 0.05, Fig. 2E, F).

### Effect of STAT3 inhibitor S3I-201 on the EMT and the level of HIF-1α in mesothelial cells induced by high glucose

**Fig. 3:**
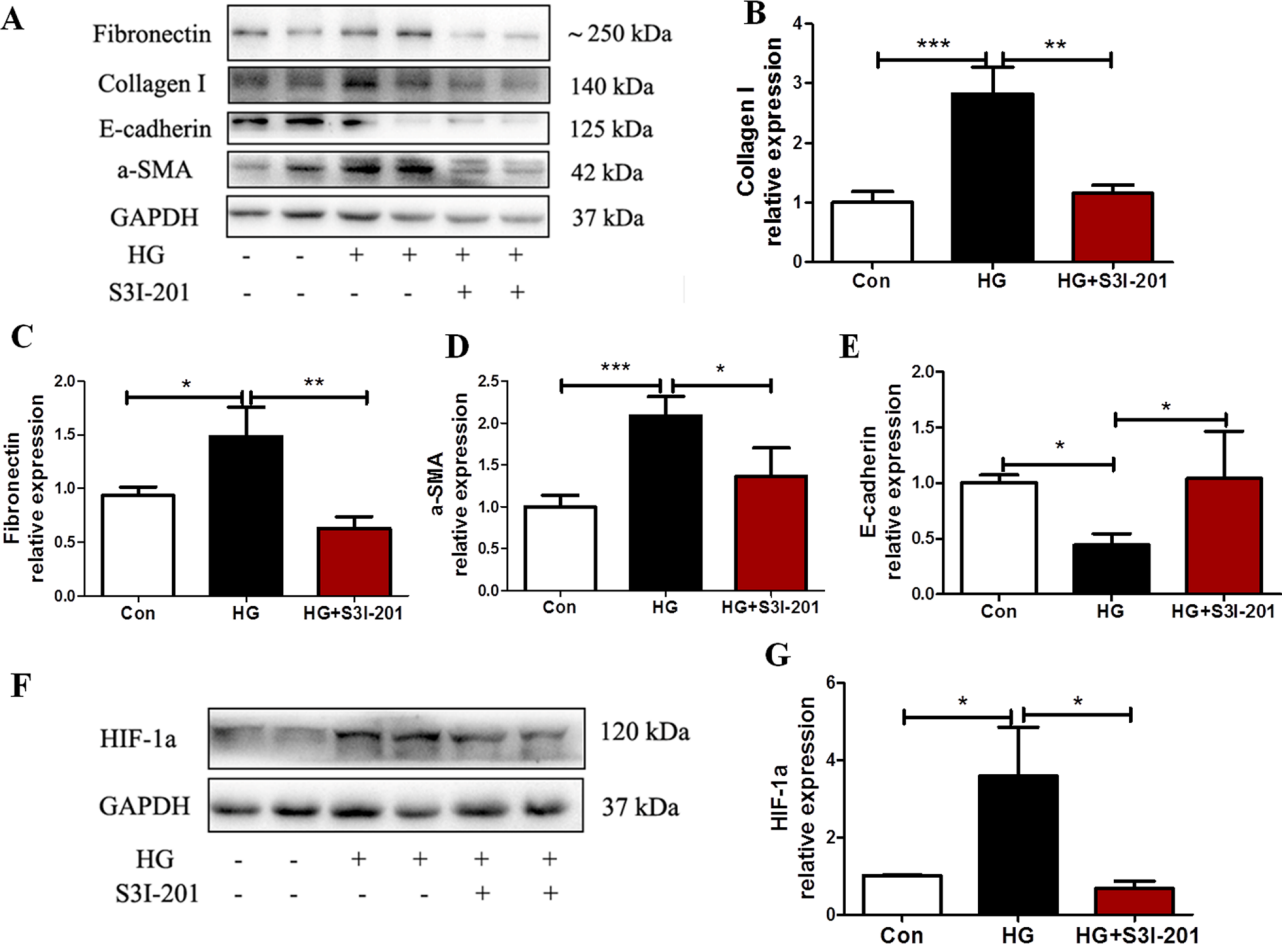
STAT3 inhibitor S3I-201 reduced Met-5A cells EMT and HIF-1α expression induced by high glucose. Met-5A cells without treatment, treated with high glucose (60 mmol/L) or in combination with S3I-201 (10 μM, pretreated for 1 h) for 48 h. **A**–**E** Expression of E-cadherin, α-SMA, Collogen I and Fibronectin as well as (**F**, **G**) expression of HIF-1α in cells by western blot analysis. GAPDH was used as a loading control. The data were the means ± SE of three experiments. *P* values were determined by t tests. *P < 0.05; **P < 0.01; ***P < 0.001; ns. not significant

Cells were incubated with 60 mmol/L high glucose for 48 h, with or without S3I-201 (10 μM, pretreatment for 1 h). We determined the subcellular location and expression of the epithelial-to-epithelial adhesion protein marker E-cadherin, the mesenchymal marker α-SMA, and the fibrosis markers Fibronectin and collagen type I by immunofluorescence (Additional file 2: Figure S1) or by western blot analysis (Fig. 3). Treatment of Met-5A cells with high glucose increased the expression of α-SMA, Fibronectin and Collagen I, but significantly decreased that of E-cadherin. The effect was attenuated by pretreatment with S3I-201 (all *P* < 0.05 compared to HG, Fig. 3A–E). Furthermore, high glucose significantly increased the expression of HIF-1α in Met-5A cells (*P* < 0.05 compared to control), which could also be reversed by pretreatment with S3I-201 (*P* < 0.05 compared to HG, Fig. 3F, G).

### Effect of STAT3 siRNA on the EMT and the level of HIF-1α in mesothelial cells induced by high glucose

**Fig. 4:**
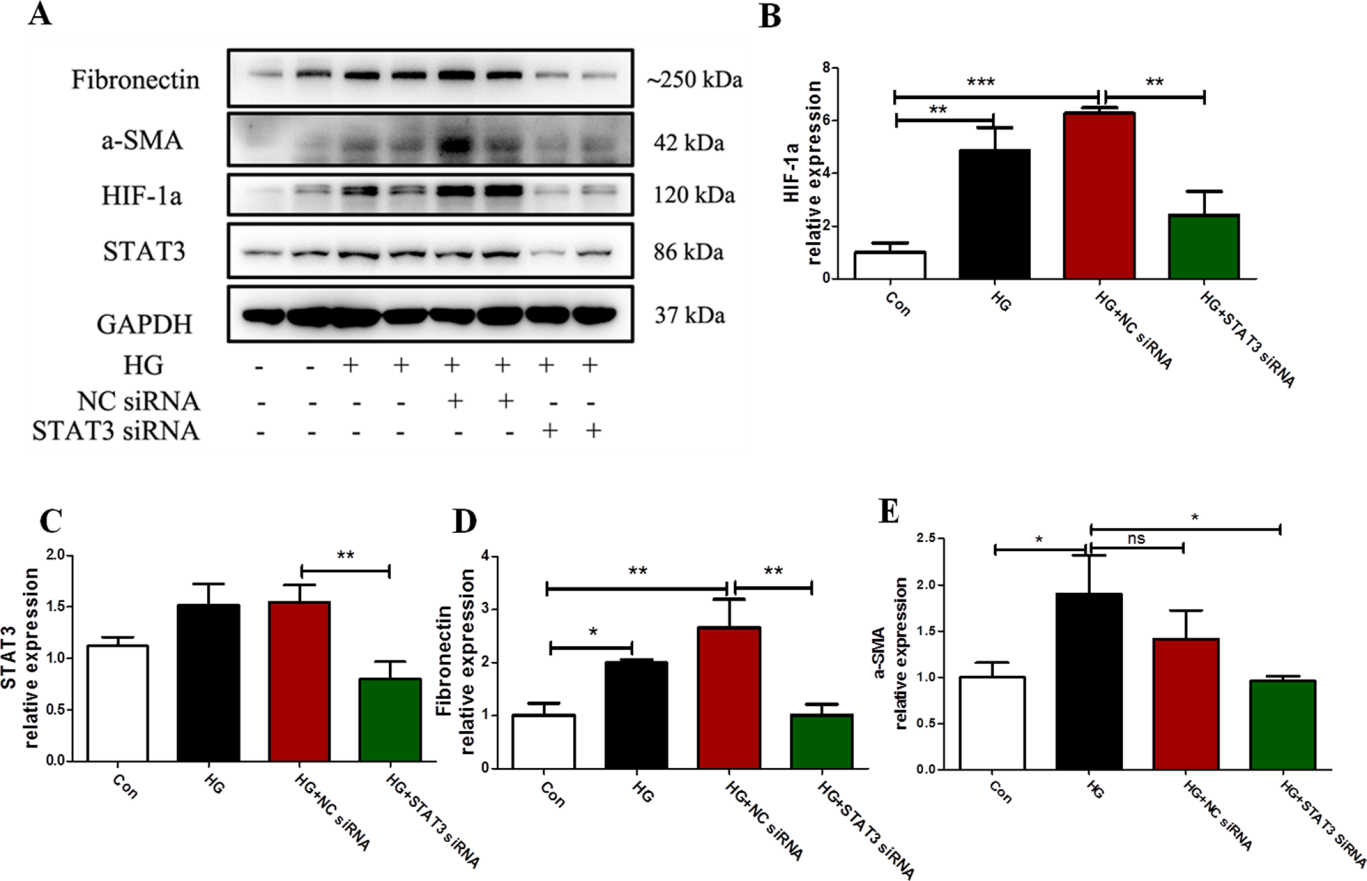
Knockdown of STAT3 alleviated Met-5A cells EMT and HIF-1α expression induced by high glucose. (A) Met-5A cells were seeded and then transfected with siRNA against STAT3 (100 nM) or negative control (100 nM) and 12 h later, cells were incubated with high glucose (60 mmol/L) for 48 h. (B-E) Expression of HIF-1α, Fibronectin, α-SMA, STAT3 in cells by western blot analysis. GAPDH was used as a loading control. The data were the means ± SE of three experiments. *P* values were determined by t tests. *P < 0.05; **P < 0.01; ***P < 0.001; ns: not significant

We analyzed the effect of high glucose on the expression of HIF-1α as well as the EMT process of mesothelial cells after transfection with siRNA against STAT3 (Fig. 4). Immunoblot analysis showed that the HIF-1α, Fibronectin, α-SMA protein levels significantly increased in high glucose induced mesothelial cells (all *P* < 0.05 compared to control, Fig. 4), which were markedly attenuated after transfection with siRNA for STAT3 (all *P* < 0.05 compared to HG or HG + NC siRNA, Fig. 4).

### Effect of HIF-1α siRNA on the EMT process of mesothelial cells induced by high glucose

**Fig. 5:**
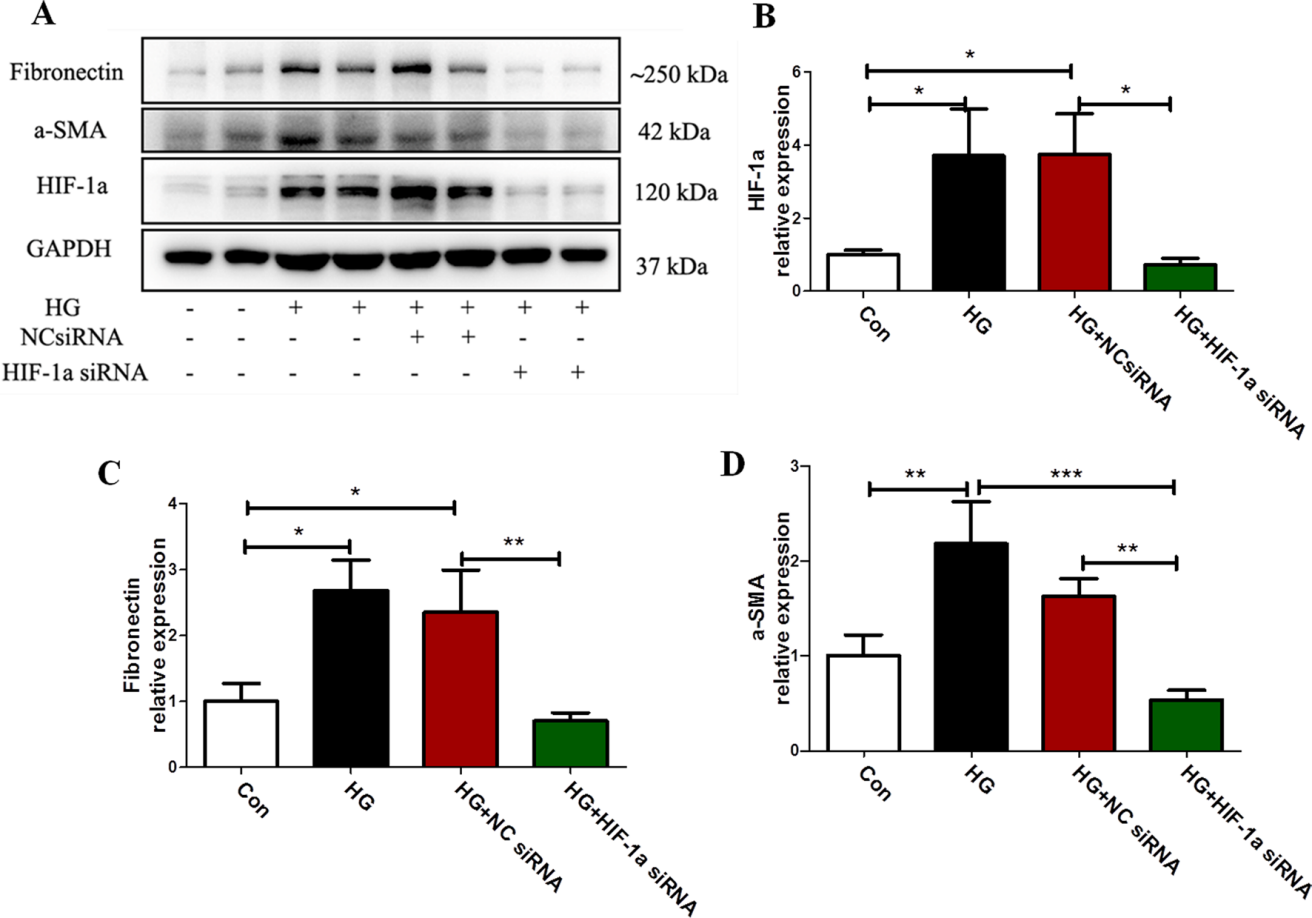
Knockdown of HIF-1α alleviated Met-5A cells EMT induced by high glucose. **A** Met-5A cells were seeded and then transfected with siRNA against HIF-1α (100 nM) or negative control (100 nM) and 12 h later, cells were incubated with high glucose (60 mmol/L) for 48 h. **B**–**D** Expression of HIF-1α, Fibronectin, α-SMA in cells by western blot analysis. GAPDH was used as a loading control. The data were the means ± SE of three experiments. *P* values were determined by t tests. *P < 0.05; **P < 0.01; ***P < 0.001

We further analyzed the effect of HIF-1α siRNA on the EMT process of mesothelial cells induced by high glucose (Fig. 5). Immunoblot analysis showed that the Fibronectin, α-SMA protein levels significantly increased in high glucose-induced mesothelial cells (all p < 0.05 compared to control, Fig. 5), which were markedly attenuated after transfection with siRNA for HIF-1α (all*P* < 0.05 compared to HG + NC siRNA, Fig. 5).

### The activation of STAT3 pathway in mouse PF model

**Fig. 6:**
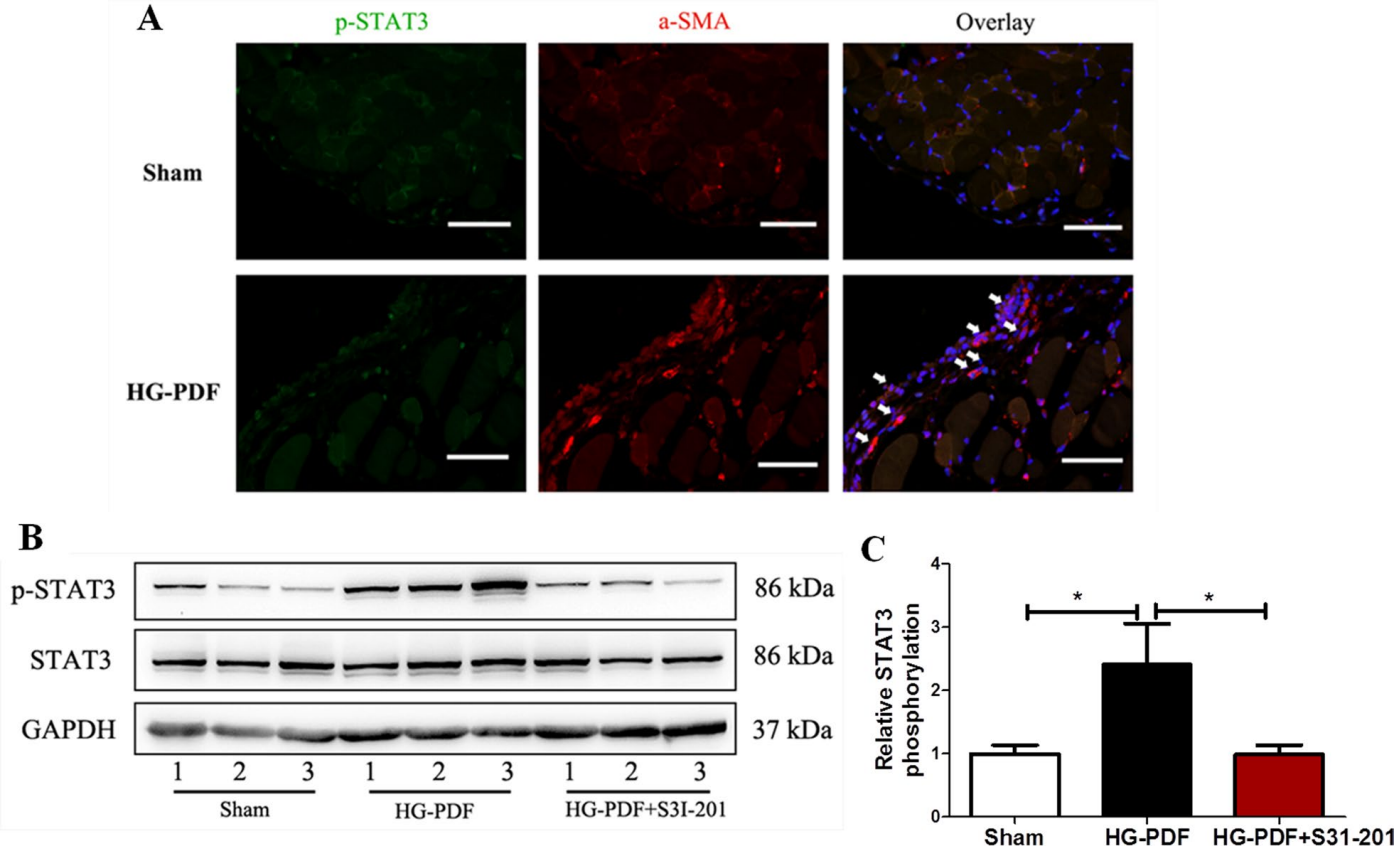
The activation of STAT3 pathway in mouse PF model. Peritoneal membrane of mice was collected at 28 days after 4.25% HG-PDF injection with or without administration of S31-201 (10 mg/kg/day). **A** Photomicrographs illustrated containing of p-STAT3 (green) and α-SMA (red) in the peritoneal membrane. Co-location of p-STAT3 (green) and α-SMA (red) in the peritoneal membrane was showed as white arrows. DAPI, 4^′^6-diamidino-2-phenylindole. Original magnification, ×200. **B**, **C** Peritoneum lysates were subjected to immunoblot analysis with specific antibodies against p-STAT3, STAT3, or GAPDH; the representative results with three samples were shown. and the relative ratios of p-STAT3 to total STAT3 were expressed as the mean ± SE of three experiments. *P* values were determined by t tests. *P < 0.05. Scale bar, 50 μm

To examine STAT3 activation in mouse PF model, we detected the expression and location of p-STAT3 in the peritoneal membrane by immunostaining and immunoblot analyses after daily injection of HG-PDF (4.25% glucose dialysis solution, Dianeal Baxter) for 28 days. Immunostaining showed that p-STAT3 was expressed mostly in α-SMA positive cells in the peritoneal membrane as described in our previous study [14] (Fig. 6A). Immunoblot analysis showed that STAT3 phosphorylation in the peritoneum of mouse PF model, while simultaneous administration of S3I-201(10 mg/kg/day) inhibited this change (*P* < 0.05 compared to HG-PDF group, Fig. 6B, C).

### In vivo effect of STAT3 inhibitor S3I-201 in mouse PF model

**Fig. 7:**
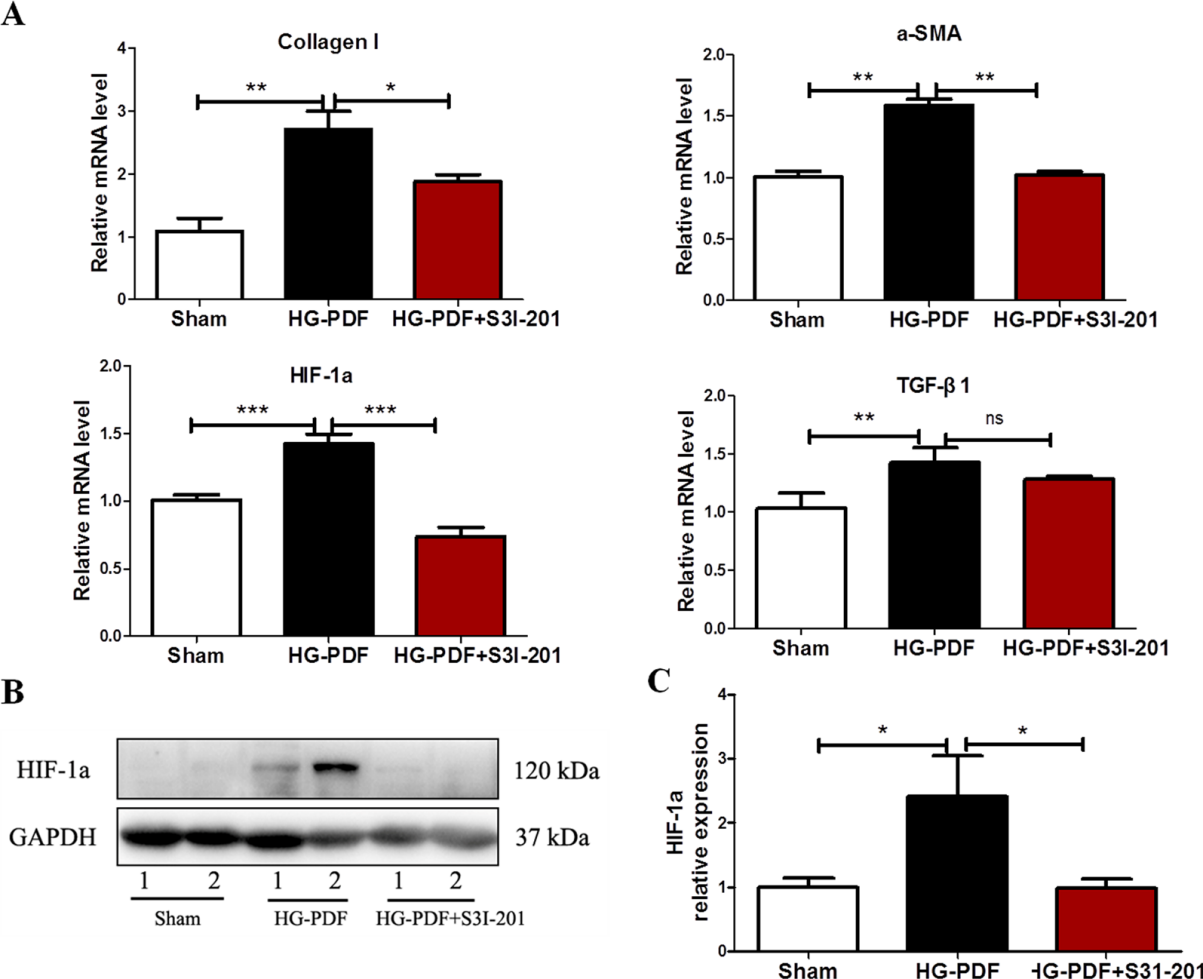
Effect of STAT3 phosphorylation blockade on level of HIF-1α and fibrosis related proteins in peritoneum. Peritoneal membrane of mice was collected at 28 days after 4.25% HG-PDF injection with or without administration of S3I-201 (10 mg/kg/day). **A** Peritoneal membrane lysates were subjected to examine the mRNA of Collagen I, α-SMA, HIF-1α and TGF-β1 using real-time PCR. **B**, **C** Peritoneal membrane lysates were subjected to immunoblot analysis with specific antibodies against HIF-1α and GAPDH; the representative results with three samples were shown. Data were expressed as the mean ± SE of three experiments. *P* values were determined by t tests. *P < 0.05; **P < 0.01; ***P < 0.001; ns: not significant

We next explored the mRNA levels of Collagen I, α-SMA, TGF-β1 as well as HIF-1α in the peritoneum of mouse PF model using real-time PCR. In the peritoneal membrane induced by HG-PDF, Collagen I, α-SMA and HIF-1α mRNA were significantly up-regulated, which was remarkably reduced after application of S3I-201(10 mg/kg/day) (*P* < 0.05 compared to HG-PDF group, Fig. 7A). TGF-β1 mRNA was also increased in mouse PF model, but had no change when treated with S3I-201(Fig. 7A). Immunoblot analysis showed that the expression of HIF-1α significantly increased in peritoneal membrane induced by HG-PDF, while simultaneous administration of S3I-201 markedly inhibited this change (p < 0.05 compared to HG-PDF group, Fig. 7B, C).

### In vivo effect of STAT3 inhibitor S3I-201 on the development of peritoneal fibrosis.

**Fig. 8:**
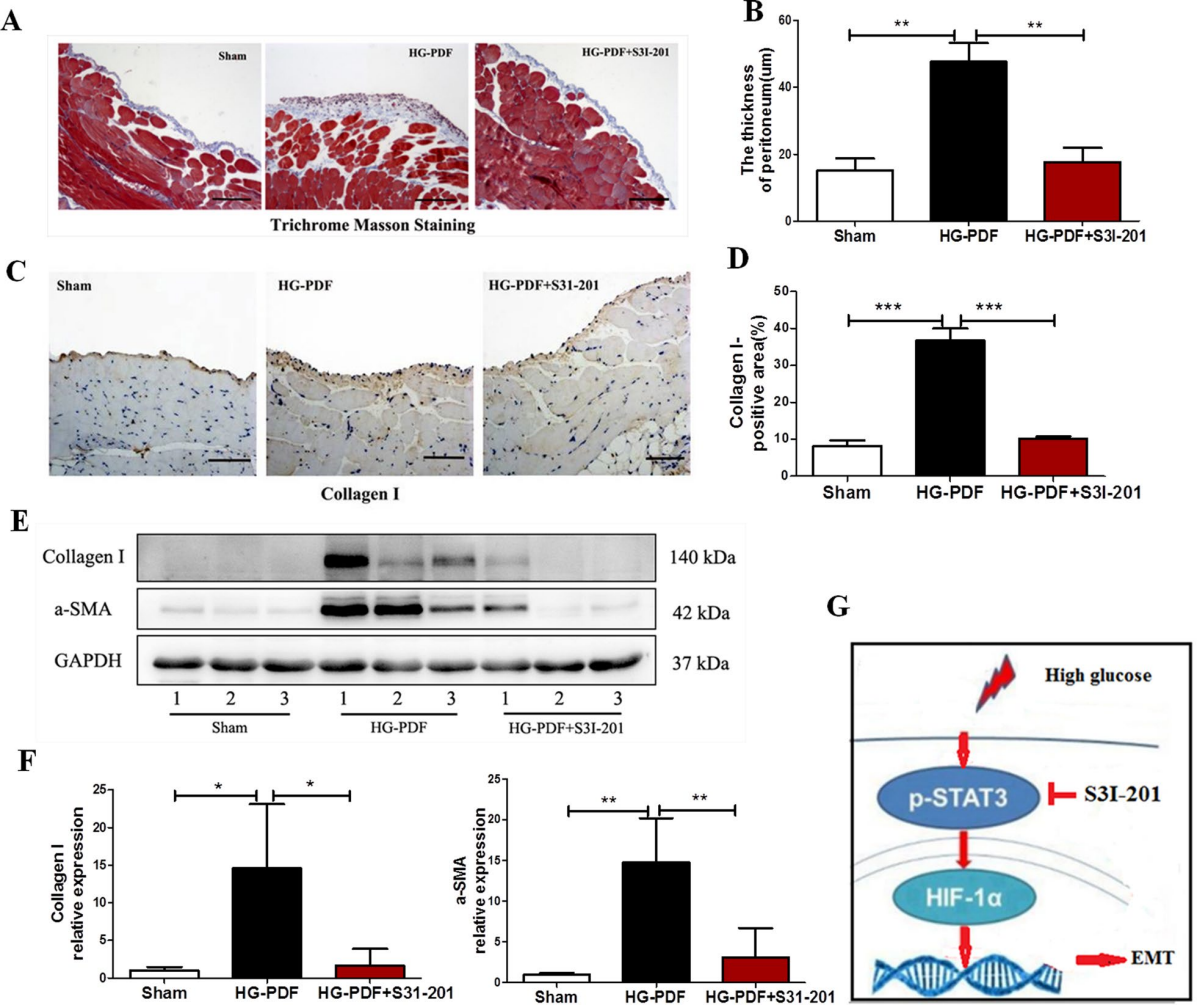
Effect of STAT3 phosphorylation blockade on the development of peritoneal fibrosis. Peritoneal membrane of mice was collected at 28 days after 4.25% HG-PDF injection with or without administration of S3I-201 (10 mg/kg/day). **A** Photomicrographs illustrated Masson trichrome stain of peritoneal membrane. Original magnification, ×200. **B** The graph showed the thickness of the compact zone measured from ten random field (original magnification, ×200) of three mice peritoneal samples. **C** Photomicrographs illustrated immunohistochemical staining of Collagen I in the submesothelial compact zone, and **D** quantitative data of relative Collagen I positive area (n = 3 per group). Original magnification, ×200. **E**, **F** Peritoneal membrane lysates were subjected to immunoblot analysis with specific antibodies against Collagen I, α-SMA, or GAPDH; the representative results with three samples were shown. Data were expressed as the mean ± SE of three experiments. *P* values were determined by t tests. *P < 0.05; **P < 0.01; ***P < 0.001. **G** Schematic of the role of STAT3/HIF-1α in peritoneal fibrosis as well as the role of STAT3 inhibitor S3I-201. Scale bar, 50 μm

We further examined the effect of STAT3 inhibitor S3I-201 on peritoneal fibrosis induced by HG-PDF in mice. Daily injection of HG-PDF for 28 days induced thickening of peritoneal membrane and deposition of collagen fibrils, as evidenced by Masson trichrome staining (Fig. 8A, B) or immunostaining (Fig. 8C, D). However, application of S3I-201 attenuated these changes. S3I-201 was also effective in suppressing the expression of collagen type I and α-SMA in the peritoneal membrane induced by HG-PDF using immunoblot analysis (*P* < 0.05 compared to HG-PDF group, Fig. 8E, F).

Increased expression of inflammatory cytokines/chemokines and infiltration of macrophages in the thickened sub-mesothelial zone were regarded as typical pathological changes in fibrotic peritoneal membrane. To determine the effect of STAT3 inhibitor on these responses after HG-PDF injury, we examined the expression of F4/80, a marker of macrophage infiltration, by immunohistochemistry. Macrophage infiltration dramatically increased in the sub-mesothelial layer of mice peritoneal membrane after HG-PDF injury but was significantly blocked by S3I-201 treatment (Additional file 3: Figure S2A, B). Next we further explored the levels of inflammatory factors including monocyte chemoattractant protein-1 (MCP-1) and interleukin-1β (IL-1β) in peritoneal membrane using real-time PCR. Both MCP-1 and IL-1β mRNA were significantly up-regulated in the HG-PDF-induced peritoneal membrane, which were remarkably reduced after application of S3I-201 (*P* < 0.05 compared to HG-PDF group, Additional file 3: Figure S2C). Moreover, the number of CD31 + vessels in the peritoneal membrane markedly increased after HG-PDF injection compared with the sham group. Administration of S3I-201 significantly suppressed the increase of blood vessels in the peritoneal membrane induced by HG-PDF (Additional file 3: Figure S2D, E).

Taken together, our data supported that HG-PDF promoted the STAT3 pathway activation and HIF-1α expression. Furthermore, the expression of STAT3/HIF-1α signaling might underlay the EMT process of mesothelial cells so as to cause peritoneal fibrosis, while STAT3 blockade might be an effective therapy for PF in long-term PD patients (Fig. 8G).

## Discussion

This study has shown that STAT3 signaling was activated in the mouse PF model induced by HG-PDF and in the HG-stimulated mesothelial cells as well as the cast-off HPMCs from drained PDF. The level of HIF-1α was up-regulated by STAT3 activation. We have certified that the HG/STAT3/HIF-1α signaling pathway might play an important role in the pathogenesis of peritoneal fibrosis induced by high-glucose based dialysis fluid. Administration of STAT3 inhibitor, S3I-201, could reduce the expression of HIF-1α and prevent the EMT of mesothelial cells and finally alleviate the development of PF. This study provided the first evidence that pharmacological inhibition of STAT3 prevented peritoneal fibrosis through the HG/STAT3/HIF-1α signaling pathway, indicating STAT3 inhibitor might be an effective therapeutic strategy for PF in PD patients undergoing long-term treatment.

Successful PD therapy largely depended on the preservation of peritoneal membrane. However, long-term PD treatment was accompanied by advanced structural and functional alterations of peritoneal membrane [27]. Presently, the mechanism leading to PF was not well understood and there was no effective therapeutic intervention for this complication. Our data indicated that STAT3 was activated in the cast-off HPMCs of drained PDF from long-term PD patients. Furthermore, we found that HG induced the phosphorylation of STAT3 as well as the expression of α-SMA, Collagen I and Fibronectin but reducing the expression of E-cadherin in human mesothelial cells, known as the EMT process. While the STAT3 inhibitor prevented the profibrotic activity in mesothelial cells and tissue accumulation of collagen in peritoneal membrane induced by high glucose. To date, activation of STAT3 signaling in peritoneal fibrosis had been reported only in some studies [14, 28, 29], activation of STAT3 signaling in the mesothelial cells induced by high glucose has been reported only in one study [16], but the role of STAT3 inhibitor in the pathogenesis of peritoneal fibrosis and potential molecules involved were still not fully understood. Our study supported that the STAT3 signaling appeared to be a mechanism by which mesothelial cells regulated profibrotic activity, contributing to the overall progression of PF and HIF-1α might be a critical molecular underlying the profibrotic role of STAT3 signaling activation (Fig. 8G).

Hypoxia contributed to the EMT of different kinds of cells, such as malignant cells and renal tubular cells [30, 31]. Morishita et al. [20] had reported that hypoxia induced the EMT of mesothelial cells via the activation of HIF-1α, which might contribute to peritoneal fibrosis in PD patients. As an important supplement, our study indicated that HIF-1α expression in mesothelial cells induced by HG was mainly dependent on STAT3 signaling activation while knockdown of HIF-1α could alleviate the EMT of mesothelial cells induced by high glucose. We also observed that STAT3 activation and HIF-1α expression were elevated in the peritoneum of mouse PF model. Given that TGF-β1 had been reported as an important contributor to the process of PF. In our study, there was also increasing expression of TGF-β1 in the fibrotic peritoneum, but its level could not be reduced by STAT3 inhibitor. Our findings suggested that the HG/STAT3/HIF-1α signaling pathway might be an important supplement for the mechanism of PF during long-term PD treatment and STAT3 inhibitor could be a new therapeutic strategy for the PF.

Increasing evidences supported that peritoneal membrane was a chronically inflamed organ during long-term PD treatment. There was a vital role of intraperitoneal inflammation in the pathogenesis of PF [14]. In our study, macrophages accumulated in the sub-mesothelial zone after injury, and blockage of STAT3 inhibited infiltration of macrophages together with attenuation of peritoneal fibrosis in mice. Inflammatory factors including MCP-1, IL-1β were also up-regulated in the HG-PDF-induced peritoneal membrane, which was also attenuated by S3I-201 in our study. A correlation between peritoneal vascular density and peritoneal fibrosis had been observed in patients undergoing long-term PD [3]. In support of this notion, we demonstrated that vascular vessels increased in the thicken sub-mesothelial zone of HG-PDF-treated mice, whereas administration of S3I-201 significantly reduced vascular density. Therefore, STAT3 pathway activation might also be involved in angiogenesis and infiltration of inflammatory cells, together with its profibrotic function, so as to play an extensive role in structural and functional alterations in the peritoneal membrane.

To date, there was no established treatment for structural alterations in peritoneal membrane. Many therapeutic interventions had been investigated with the aim to prevent some of the major pathological processes, such as the fibroblast activation, inflammation, and angiogenesis, involved in peritoneal fibrosis [32, 33]. Our study indicated that the HG/STAT3/HIF-1α signaling pathway might involve in all those processes and the treatment using STAT3 inhibitor could partly attenuate the progression of peritoneal fibrosis, angiogenesis and inflammation.

Our study had some limitations. We used intraperitoneal injection rather than peritoneal catheter instillation of high glucose-based dialysate to induce peritoneal fibrosis in mice, although several studies had shown that direct intraperitoneal injection could induce peritoneal fibrosis in mice and resemble structural alterations of the peritoneal membrane in patients undergoing long-term PD treatment [29, 34, 35]. Secondly, we used immortalized human pleural mesothelial cells (Met-5A) rather than primary human peritoneal mesothelial cells, although several studies had shown that Met-5A cells phenotypically and functionally resemble normal human peritoneal mesothelial cells [22, 36, 37]. A recent review suggested that the data obtained from Met-5A cells had shown much concordance with data obtained from primary peritoneal mesothelial cells [37]. Finally, due to quite a few number of patients included in our study, a further study with a sufficient number of PD patients are required to confirm the relationship between STAT3 phosphorylation and PD duration.

In conclusion, findings in our study provided initial and important insight into the role of STAT3 signaling in mediating the EMT of mesothelial cells through up-regulating the expression of HIF-1α. S3I-201effectively attenuated the EMT of mesothelial cells induced by high glucose and ultimately alleviated the progression of peritoneal fibrosis. Thus, targeting STAT3/ HIF-1α signaling might be an effective approach to preserve the integrity of peritoneum in patients during long-term PD treatment.

## Supplementary Information

**Additional file 1: Table S1.** Clinical characteristics of patients undergoing PD.

**Additional file 2: Figure S1.** High glucose induced the EMT of mesothelial cells. Mesothelial cells without treatment, treated with high glucose (60 mmol/L) or in combination with S3I-201(10 μM, pretreated for 1 h) for 48 h. (A) Subcellular localization of E-cadherin, α-SMA, Fibronectin and Collagen I in mesothelial cells were detected by immunofluorescence analysis, and (B) quantitative data of relative fluorescence intensity (n = 3 per group). E-cadherin and Fibronectin were detected using Alex Fluor^@^488-conjugated secondary antibody (green), while α-SMA and Collagen I using Alex Fluor^@^ 594-conjugated secondary antibody (red). Original magnification, ×200. *P < 0.05; *** *P* < 0.001.

**Additional file 3: Figure S2.** Effect of STAT3 phosphorylation blockade on inflammation and angiogenesis in the peritoneal membrane. Peritoneal membrane of mice was collected at 28 days after 4.25% HG-PDF injection with or without administration of S3I-201 (10 mg/kg/day). (A) Photomicrographs illustrated immunohistochemical staining of F4/80 in the submesothelial compact zone, and (B) quantitative data of relative F4/80 positive area (n = 3 per group). Original magnification,  ×200. (C) Peritoneal membrane lysates were subjected to examine the levels of MCP-1 and IL-1β mRNA using real-time PCR. Results were displayed as mean ± SE (n = 4). (D) Photomicrographs illustrated immunohistochemical staining of CD31 in the submesothelial compact zone, and (E) quantitative data of relative CD31 positive area (n = 3 per group). Results were displayed as mean ± SE. Original magnification, ×200. *P* values were determined by t tests. ** *P* < 0.01; *** *P* < 0.001. Scale bar, 50 μm.

## Data Availability

All data generated or analyzed during this study are included in this published article (and its Additional files).

## Abbreviations

EMT: Epithelial-mesenchymal transition
PF: Peritoneal fibrosis
STAT3: Signal transducer and activator of transcription 3
HIF-1α: Hypoxia inducible factor-1α
PD: Peritoneal dialysis
PDF: Peritoneal dialysis fluid
HG-PDF: High-glucose based dialysis fluid
ESRD: End-stage renal disease
α-SMA: α-smooth muscle actin
HPMCs: Human peritoneal mesothelial cells
PSTR: Peritoneal small-solute transport rate
IL-6: Interleukin-6
TGF-β1: Transforming growth factor-β1
ACE: Angiotensin II generating enzyme
AT1: Angiotensin II receptor type 1
VEGF: Vascular endothelial cell growth factor
IL-37: Interleukin-37
CKD: Chronic kidney disease
PBS: Phosphate-buffered saline
NC: Negative control
SE: Standard error
MCP-1: Monocyte chemoattractant protein-1
IL-1β: Interleukin-1β
