# Supplementary material for: STAT3/HIF-1α signaling activation mediates peritoneal fibrosis induced by high glucose
Source: J Transl Med. 2021 Jun 30;19:283. doi: 10.1186/s12967-021-02946-8 (PMC8246671; doi:10.1186/s12967-021-02946-8)
Supplement: Supplementary file 1 — Additional file 1: Table S1. Clinical characteristics of patients undergoing PD. [file 12967_2021_2946_MOESM1_ESM.docx]

Table S1 Clinical characteristics of patients undergoing PD

| Patient  No. | Sex | Age, yr | End-Stage Renal  Disease Etiology | PD duration |
| --- | --- | --- | --- | --- |
| 1 | male | 53 | Unclear etiology | 1 month |
| 2 | male | 65 | Diabetes Nephrology | 1 year |
| 3 | male | 63 | Unclear etiology | 3 years |
| 4 | female | 63 | Chronic glomerulonephritis | 6 years |
