# Supplementary figures and images for: STAT3/HIF-1α signaling activation mediates peritoneal fibrosis induced by high glucose
Source: J Transl Med. 2021 Jun 30;19:283. doi: 10.1186/s12967-021-02946-8 (PMC8246671; doi:10.1186/s12967-021-02946-8)

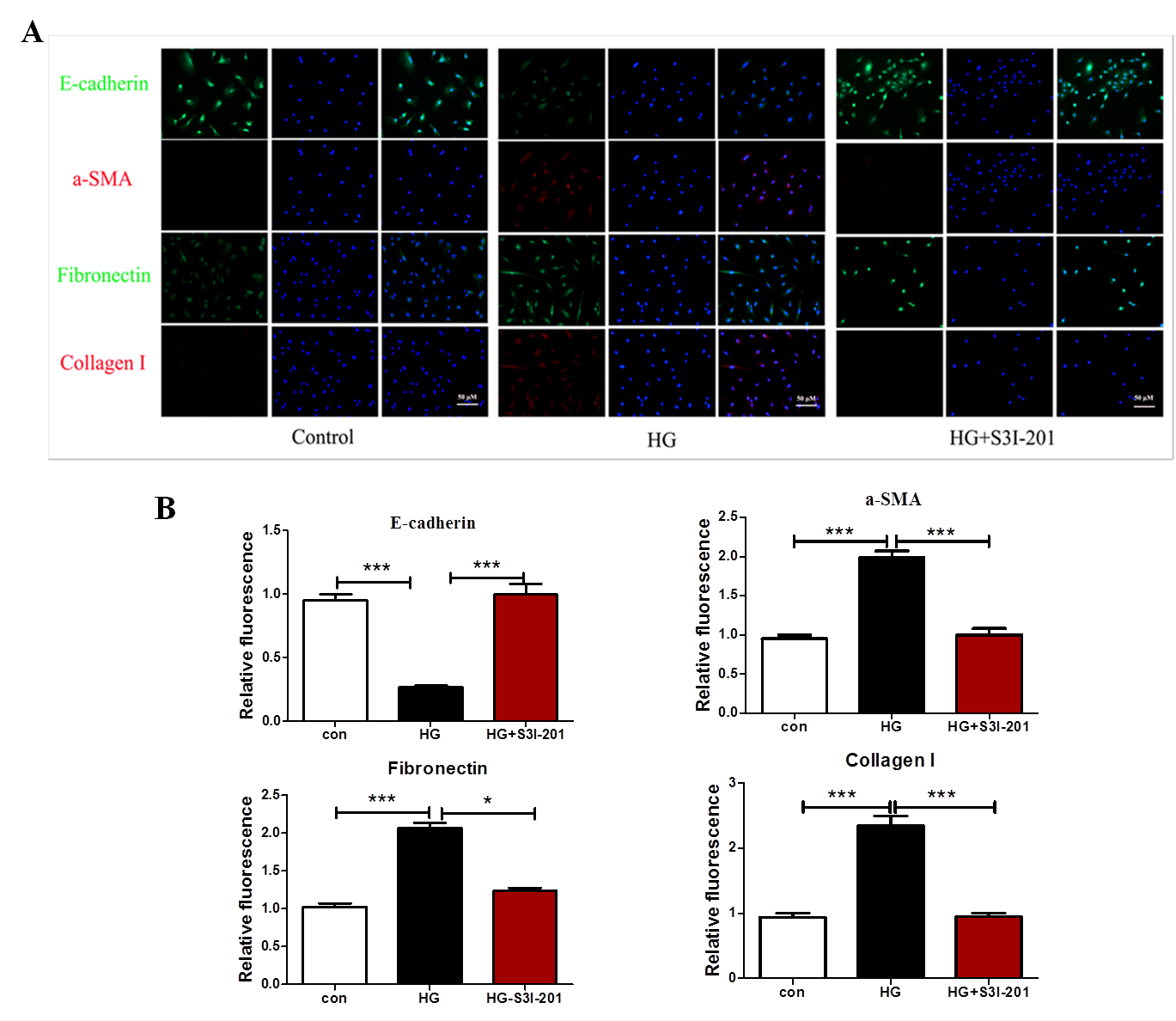

Supplement: Supplementary file 2 — Additional file 2: Figure S1. High glucose induced the EMT of mesothelial cells. Mesothelial cells without treatment, treated with high glucose (60 mmol/L) or in combination with S3I-201(10 μM, pretreated for 1 h) for 48 h. (A) Subcellular localization of E-cadherin, α-SMA, Fibronectin and Collagen I in mesothelial cells were detected by immunofluorescence analysis, and (B) quantitative data of relative fluorescence intensity (n = 3 per group). E-cadherin and Fibronectin were detected using Alex Fluor@488-conjugated secondary antibody (green), while α-SMA and Collagen I using Alex Fluor@ 594-conjugated secondary antibody (red). Original magnification, ×200. *P < 0.05; *** P < 0.001. [file 12967_2021_2946_MOESM2_ESM.png]

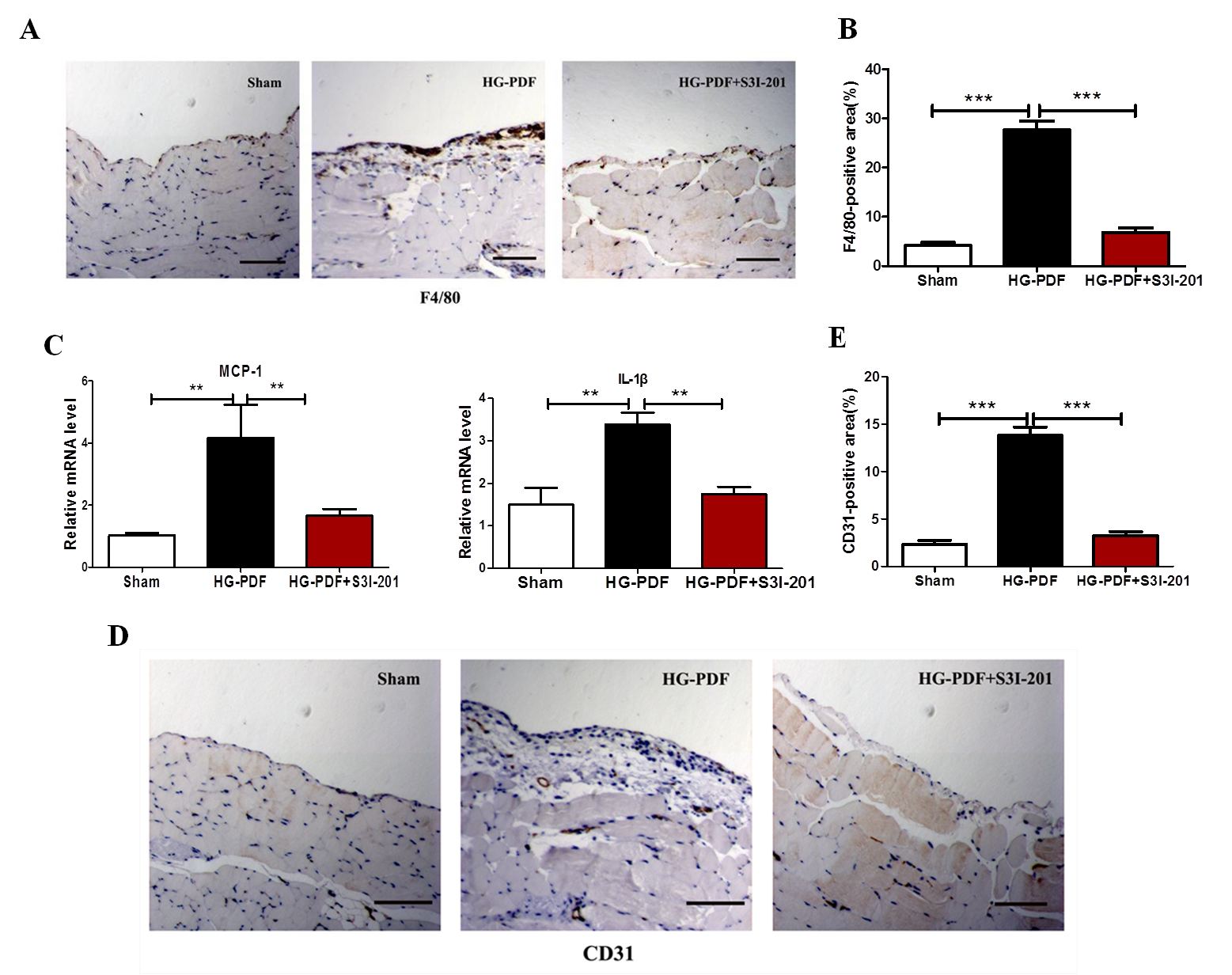

Supplement: Supplementary file 3 — Additional file 3: Figure S2. Effect of STAT3 phosphorylation blockade on inflammation and angiogenesis in the peritoneal membrane. Peritoneal membrane of mice was collected at 28 days after 4.25% HG-PDF injection with or without administration of S3I-201 (10 mg/kg/day). (A) Photomicrographs illustrated immunohistochemical staining of F4/80 in the submesothelial compact zone, and (B) quantitative data of relative F4/80 positive area (n = 3 per group). Original magnification, ×200. (C) Peritoneal membrane lysates were subjected to examine the levels of MCP-1 and IL-1β mRNA using real-time PCR. Results were displayed as mean ± SE (n = 4). (D) Photomicrographs illustrated immunohistochemical staining of CD31 in the submesothelial compact zone, and (E) quantitative data of relative CD31 positive area (n = 3 per group). Results were displayed as mean ± SE. Original magnification, ×200. P values were determined by t tests. ** P < 0.01; *** P < 0.001. Scale bar, 50 μm. [file 12967_2021_2946_MOESM3_ESM.png]
